# Laboratory evaluation of the potential masking of color changes produced by silver diamine fluoride in primary molars

**DOI:** 10.1186/s12903-021-01697-8

**Published:** 2021-07-09

**Authors:** Dina Hamdy, Maria Giraki, Amr Abd Elaziz, Amira Badran, Gehan Allam, Stefan Ruettermann

**Affiliations:** 1 Department of Pediatric Dentistry and Dental Public Health, Faculty of Dentistry, Ain Shams University, Organization of African Unity Street, El Qobba Bridge, Al Waili, Cairo, 11865 Egypt; 2 Department of Operative Dentistry, Dental School (Carolinum), Goethe-University Frankfurt, Theodor-Stern-Kai 7/29, 60596 Frankfurt am Main, Germany

**Keywords:** Silver diamine fluoride, Primary molars, Potassium iodide, Masking effect, COVID-19 pandemic, Minimal invasive dentistry, Nonaerosolizing treatment

## Abstract

**Background:**

The importance of Silver diamine fluoride (SDF) as a minimally invasive and nonaerosolizing management during COVID-19 pandemic has highly increased. SDF is a caries-arresting agent that causes staining of tooth structure. Managing this discoloration will increase its acceptance in treating primary teeth. The main aim of this study was to quantify the color change associated with the application of SDF on extracted carious primary molars, the potential masking of this color change by potassium iodide (KI), composite (CMP) and glass ionomer cement (GI) and the effect of aging on this color masking effect.

**Methods:**

An in-vitro study in which 52 carious primary molars were collected, prepared, and distributed randomly into four groups equally as follows: Group A: SDF 38%; Group B: SDF 38% + KI; Group C: SDF 38% + CMP; Group D: SDF 38% + GI. Color changes were recorded for each sample at baseline, and after application of the tested materials. Moreover, all samples had undergone Suntest aging followed by a third color reading. CIELAB values L*, a*, b*, ΔL, Δa, and Δb were measured, ΔE was calculated, and data were analyzed using multivariate analysis of variance (MANOVA) and post-Hoc Scheffé test (*p* < 0.05).

**Results:**

MANOVA revealed the significant influence of the factor ‘material’. SDF caused an obvious color change compared to the color of carious dentin. Regarding ΔL, the color change of groups C and D was not significant directly after application of the tested materials. After aging, it was significant among all groups, including groups C and D. In Δa there was a difference between SDF and groups B and C after application of the tested materials, and aging produced the same results. The color shifts of Δb of all tested groups varied significantly from one another. After aging, there was no difference between group D and either group A or B.

**Conclusions:**

Treatment with SDF caused obvious discoloration of carious dentin. Directly after SDF application, all tested materials could effectively mask the color change associated with the application of SDF. CMP was the only material whose color masking effect was not completely reversed by aging.

## Background

The treatment of carious lesions has changed over time, and conventional approaches involving total removal of the decayed tissue have been replaced by minimally invasive approaches aiming to mainly prevent disease progression and preserve pulp vitality [1]. However, the development of cost-effective caries prevention and arresting techniques is still a major challenge [2, 3].

Since the COVID-19 virus was announced by the World Health Organization (WHO) to be a worldwide pandemic whose transmission occurs mainly by droplet infection, which can be caused by rotary instruments in dental practice [4], dental public healthcare workers have been continuously searching for effective, simple and aerosol-free methods that maximize caries prevention while minimizing the spread of infection [5]. In addition, the use of nonsurgical/nonrestorative cavity treatment (NRCT) as emergency management for dental caries was adopted [6].

NRCT is a treatment option for dentinal caries in primary teeth in which the cavity margins will be exposed to enable good oral hygiene measures. Silver diamine fluoride (SDF) application is recommended as a support method for NRCT for cases of active caries or for patients with high caries risk [7].

NRCT can be as effective as restorations in the management of carious lesions in deciduous teeth [8, 9]. However, in some cases, restorations should still be performed after ensuring the successful arrest of the lesion for the comfort of the patient and promoting oral health until the exfoliation of the primary tooth [10].

Several approaches have been developed over the years to arrest caries and preserve pulp vitality [1], including the use of chemotherapeutics including metal ions [11], antibiotics [11], fluorides [12] and probiotics [13]. Chemotherapeutic silver preparations, including silver nitrate, SDF, and ammonium hexafluoro silicate, are also used to arrest active caries, particularly in primary teeth [14].

In 2014, SDF was approved for dental use in many countries [15]. The recent suggestion of SDF as an appropriate, nonsurgical and nonaerosolizing management paradigm for painless and painful carious lesions without pulpal involvement during the COVID-19 pandemic has prompted its further study [5].

SDF is a safe, minimally invasive, low cost, portable material that can be used in variable community settings by members of a healthcare team [5, 6]. It is known for its antibacterial effect [16], as well as its caries-arresting effect [17], and it is one of the best treatment approaches for controlling dental caries in primary dentition [18]. However, it may not eliminate the need for a restorative approach if the lesion fails to arrest or increases in size and/or depth. In this case, the main function of SDF is to slow down the progression of the disease and bridge the time gap until a definitive treatment can be administered [19].

Similarly, SDF can be used alongside the atraumatic restorative technique (ART), thus preventing irreversible pulpitis or dental infections of sealed dental lesions in a technique known as silver-modified ART (SMART) [20].

For SDF to be acceptable on a wider scale as a treatment modality for primary teeth, unfavorable adverse effects, such as staining of the tooth structure and adjacent tooth-colored restorations, should be managed [18, 21], since this is a considerable concern of patients and parents [22, 23].

Several materials have been used to mask the unfavorable tooth discoloration from SDF (for example, KI can be applied immediately after SDF), suggesting that discoloration of carious lesions can be masked without reducing the caries-arresting effect [24, 25]. However, studies quantifying tooth discoloration after SDF application, as well as the masking effect of KI [26], have been predominantly conducted on permanent teeth, as described in a recent systematic review by Roberts et al. [27].

The contraindications of KI use in patients undergoing thyroid gland therapy or in patients with allergies to potassium or iodine increases the need to evaluate other discoloration masking techniques [19]. In their review, Roberts et al. [27] found only one in vitro study comparing white restorative materials and their subsequent impacts on tooth lightness in permanent teeth [28]. However, the literature regarding the masking effect of tooth-colored restorations such as CMP and GI on the tooth discoloration associated with SDF application is still deficient, especially in primary teeth. Hence, this in vitro trial aims to quantify the color change associated with the application of SDF on extracted carious primary molars; the potential masking of this color change by KI, CMP, and GI; and the effect of aging on this color masking effect.

## Methods

### Study setting

A total of 52 extracted primary molars with occlusal caries extending to the dentin were collected from the outpatient clinic at the Department of Pediatric Dentistry and Dental Public Health, Faculty of Dentistry, Ain Shams University. All extracted teeth were nonrestorable and were collected from children between 4 and 7 years of age from both sexes with no previous history of systemic diseases.

All experimental protocols were approved by the Research Ethics Committee of the Faculty of Dentistry of Ain Shams University, Egypt (FDASUREC), under the number FDASU-RECID111610. The authors followed the institutional and international guidelines mentioned in the Declaration of Helsinki for the use of human body material in medical research. In accordance with the requirements of the Research Ethics Committee, both verbal and written informed consent were obtained from the legal guardian of the child before the collection of the extracted teeth. The methodology applied followed the checklist for reporting in vitro studies (CRIS Guidelines) [29].

The study was carried out in the Department of Operative Dentistry, Dental School (Carolinum), Goethe University Frankfurt, Germany, over a period of 6 months. This was a laboratory study in which balanced block randomization with an allocation ratio of 1:1 was used.

### Tooth preparation

First, plaque was thoroughly cleaned from the teeth using a polishing brush and a nonfluoridated prophylactic paste (Clean Polish, Kerr, Switzerland) in a low-speed handpiece (Dentsply Sirona, Germany) at a speed of 4,000 rpm. Then, the teeth were mounted in acrylic resin (Technovit® 4000, Kulzer, Germany) cylinders 3 cm in height and 1.5 cm in diameter to facilitate their cutting and handling. Afterwards, they were stored in distilled water at room temperature.

**Fig. 1:**
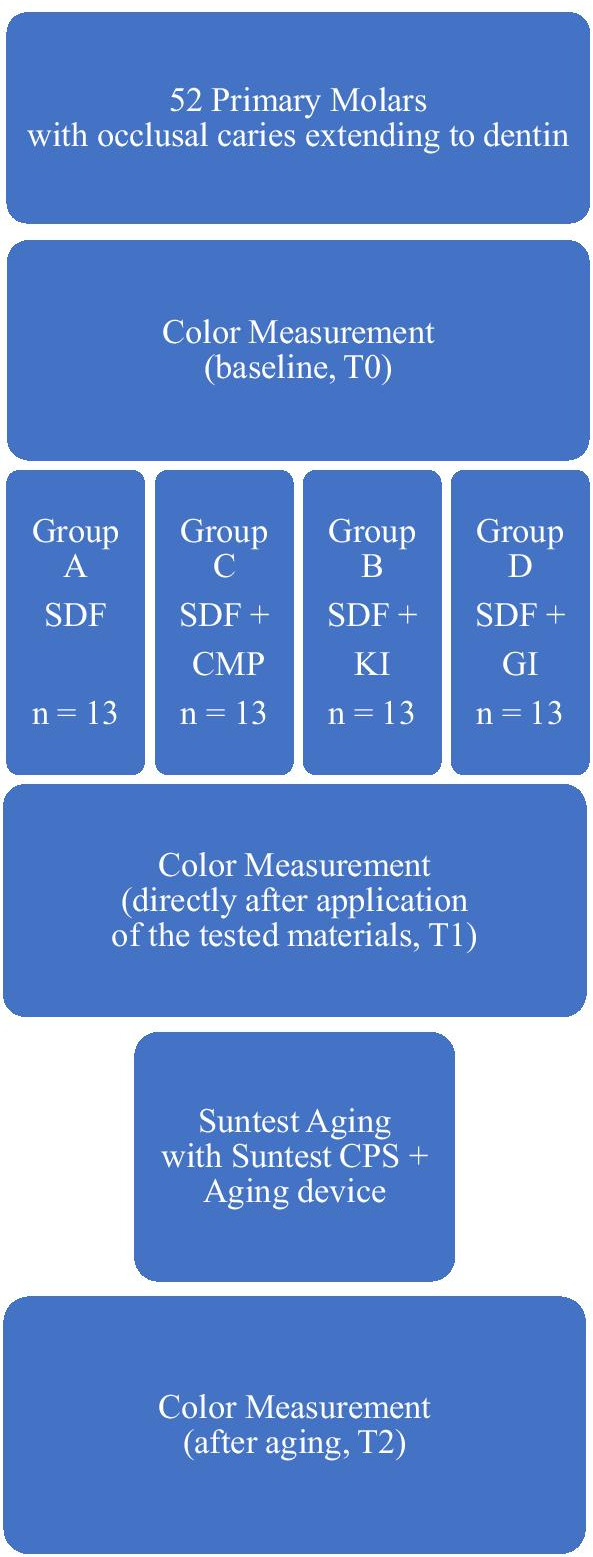
Experimental setup

The enamel surrounding a carious lesion was ground with water-cooled carborundum discs (6911 HK, Komet Dental, Germany) attached to a low-speed motor (Ultimate 450, NSK, Germany) at a speed of 6000 rpm, creating a flat occlusal surface with exposed dentin. Carious dentin slices (5 × 5 mm) were then cut out of the tooth with the same disc. To ensure size standardization of the sample, pink wax was cut into small squares (5 × 5 mm), placed over the carious lesion during cutting and then removed immediately thereafter. Molars were then randomly divided into four groups (13 teeth each) according to the materials tested: Group A, SDF 38%; Group B, SDF 38% + KI; Group C, SDF 38% + CMP; and Group D, SDF 38% + GI. Figure 1 shows the experimental setup.

### Addition of the tested materials

A baseline reading was recorded for all the samples before the addition of any of the tested materials. One drop of SDF (Riva Star, SDI GmbH**,** Germany) was applied to all specimens for 1 min, and then the specimens were rinsed with water for another 30 s and dried with air. Group B: KI was applied to the tooth directly after SDF according to the manufacturer’s instructions until the white precipitate totally disappeared. Group C: After the application and rinsing of SDF, a universal self-etching adhesive (Scotchbond™, 3 M ESPE, USA) was applied. A 4 mm layer of universal composite restorative material, shade A1 (Filtek Z250, 3 M ESPE, USA), was added in 2 increments of 2 mm each and then light cured by an LED polymerization lamp (blue phase, Ivoclar Vivadent AG, Schaan, Liechtenstein) for 20 s with a power of 1100 mw/cm^2^ and a wavelength of 500 nm. Group D: The dentin was conditioned (Dentin Conditioner, GC Corporation, Japan) for 20 s and then rinsed, and a fast-setting glass ionomer restoration, shade A1 (Fuji IX Gp, GC Corporation, Japan), was added in 4 mm increments, followed by a topcoat (G-Plus, GC Corporation, Japan), which was light cured for 20 s with the same LED polymerization lamp. The color was recorded for all specimens directly after applying the tested materials. All specimens underwent aging using a Suntest aging device (Suntest CPS + , Atlas Material Testing Technology GmbH, Linsengericht, Germany) with a xenon arc lamp (150,000 lx, wavelength > 370 nm) for 24 h in water at 37 °C according to EN ISO 7491, and the color was recorded [30].

### Color measurement

Color was recorded by a spectrophotometer (X-Rite SP62, X-Rite GmbH, Cologne, Germany). The device was adjusted on a D-65 standard illuminant and calibrated once daily on both white and black calibration tiles. Each specimen was dried and centered over the 4 mm diameter aperture of the device, and the mean of 4 recordings was taken for each reading. Three readings were recorded for each specimen as follows: 1 = baseline, 2 = directly after the application of the tested material, and 3 = after Suntest aging. The L*, a*, and b* values (L* = lightness, + a* = red, − a* = green, + b* = yellow, − b* = blue) were measured to calculate ΔL, Δa, and Δb. Then, the extent of the color change (ΔE) was assessed using the following equation [31]:

$$\Delta {\text{E}} = \surd \left( {{\text{L1}} - {\text{L2}}} \right)^{{\text{2}}} + \left( {{\text{a1}} - {\text{a2}}} \right)^{{\text{2}}} + \left( {{\text{b1}} - {\text{b2}}} \right)^{{\text{2}}}$$

### Outcome

The primary endpoint of this study was to identify and assess the ability of the factor “material” to mask the color change produced by the application of SDF on primary dentin.

The secondary endpoint of this study was to determine the direction of this color change in an attempt to better understand the masking effect and hence provide better esthetic outcomes.

### Statistical analysis

Statistical analysis was conducted with SPSS 24 (SPSS, IBM, USA). A proposed sample size of 52 primary molars (13 in each group) was considered sufficient to detect an effect size of f = 0.4 with a power of 70% with a significance level of 5% [32]. The means and standard deviations were calculated.

Samples were numbered and placed in identical containers by one operator and allocated to the different groups by a computer-generated program to create a balanced block randomization with an allocation ratio of 1:1 using random block sizes of 4 and 8 [33], while the implementation of the experiment itself was done by another operator.

The primary endpoint was analyzed using multivariate analysis of variance (MANOVA) to test the effect of the factor “material”. The Scheffé post hoc test was applied for multiple comparisons among the different treatment groups. The first comparison was among all the tested groups to evaluate the differences between T0-T1, while the second compared the tested groups to evaluate the differences between T1-T2. Statistical significance was considered at *p* < 0.05 for all tests.

In addition, a descriptive statistic including the means and standard deviations for the values L*, a*, and b* was used to interpret the direction of color change in different groups at different time points (T0, T1, and T2) to fulfill the secondary outcome.

## Results

The primary analysis included all samples that were randomized. MANOVA revealed the significant influence of the factor “material” (*p* < 0.001). SDF was reported to cause an obvious color change compared to the color of carious dentin (ΔE = 17.2) when observed by the naked eye.

As observed in Table 1, the ΔL results from T0-T1 revealed a color shift that was significantly different among all treatment groups (0.001 < *p* < 0.008) except groups C and D (*p* = 0.28). After Suntest aging, this difference was significant among all groups, including groups C and D (*p* = 0.04).

**Table 1:** ΔL, Δa, Δb of different treatment groups in respect to time

	SDF (Group A)	SDF + KI (Group B)	SDF + CMP (Group C)	SDF + GI (Group D)
Color difference between baseline reading and directly after application of the tested materials (T 0,1)				
ΔL	− 16 (7) ^a^	11.7 (6) ^b^	28.1 (9.4) ^c^	22.3 (7.1) ^c^
Δa	− 1 (1.1) ^a^	− 3.9 (1.6) ^b^	− 4.2 (1.4) ^b^	− 0.9 (1.4) ^a^
Δb	− 5.5 (2.8) ^a^	5.6 (2.9) ^b^	− 2.8 (2.2) ^c^	2.4 (1) ^d^
Color difference between directly after the application of the tested materials and after Suntest aging (T 1,2)				
ΔL	− 5.6 (2.1) ^a^	− 13 (6) ^b^	− 20.7 (5.7) ^c^	− 27 (6.6) ^d^
Δa	− 1.6 (1) ^a^	− 0.02 (1.2) ^b^	2.3 (0.7) ^c^	− 0.5 (0.9) ^a, b^
Δb	− 3.3 (2.1) ^a^	− 8.1 (3.8) ^b^	7.7 (4.4) ^c^	− 4.8 (1.7) ^a, b^

Mean values and (Standard deviations) of ΔL, Δa, Δb of all treatment groups (T0, 1 and T1, 2)

Different superscript letters indicate a significant difference between the groups within one row (*p* < 0.05)

ΔL: difference in color shift of the white/black scale between two different time points

Δa: difference in color shift of the red/green scale between two different time points

Δb: difference in color shift of the yellow/blue scale between two different time points

(T0, 1): difference between baseline, and directly after application of tested materials

(T1, 2): difference in reading between directly after application of tested materials and after aging

Regarding the red/green scale (Δa), SDF resulted in a color shift that was significantly different for groups B (*p* = 0.001) and C (*p* = 0.001). Moreover, group B showed a significant difference in color shift in comparison to group D (*p* = 0.001). A significant difference in the color shift was also observed between groups C and D (*p* = 0.001). Suntest aging produced the same results, with the exception that the color shift of group B was significantly different from that of group C (*p* = 0.001) instead of group D (*p* = 0.64).

With respect to the blue/yellow scale (Δb), the color shifts of all tested groups varied significantly from one another (with significance ranging from *p* < 0.001 to *p* < 0.04). After aging, there was no difference in color shift between group D and either group A (*p* = 0.67) or group B (*p* = 0.10).

The secondary analysis also included all randomized samples. To interpret the origin and direction of the color change, L*, a*, and b* values were calculated descriptively and summarized in Table 2. The L* values suggest that directly after application of the tested materials, SDF resulted in a tooth color that was darker than the color of carious dentin, while all treatment materials (KI, CMP, and GI) resulted in a lighter tooth color, with CMP resulting in the lightest, followed by GI and KI. Aging decreased the L* values in all the groups (shift to darker colors) compared to the values directly after material application; CMP was the only material that maintained a value corresponding to a color lighter than the tooth baseline color.

**Table 2:** L*, a*, b* values of the different treatment factors in respect to time

	SDF (Group A)			SDF + KI (Group B)			SDF + CMP (Group C)			SDF + GI (Group D)		
	T0	T1	T2	T0	T1	T2	T0	T1	T2	T0	T1	T2
L*	45.9 (8.7)	30 (3.3)	24.3 (2.9)	43 (3.8)	54.7 (6)	41.6 (4.3)	41 (6.1)	69 (7.4)	48.3 (5.3)	40 (8.4)	62.3 (7.4)	35.3 (5.8)
a*	3.6 (1.5)	2.6 (1.1)	1 (0.4)	3.2 (1.3)	− 0.7 (1.2)	− 0.6 (0.4)	3.6 (1.5)	− 0.7 (0.4)	1.6 (0.6)	2.7 (1.9)	1.8 (0.8)	1.3 (1)
b*	10.9 (4.6)	5.5 (2.2)	2.2 (0.9)	9 (2.9)	14.6 (3.5)	6.5 (2.2)	7.5 (1.6)	4.8 (2.6)	12.4 (2.9)	7.3 (1.1)	9.7 (1.7)	4.8 (2.4)

Mean values and (standard deviations) of L*, a*, and b* in different readings (T0, T1, T2)

L*: direction of color change of the white/black scale

a*: direction of color change of the red/green scale

b*: direction of color change of the blue/yellow scale

T0: reading at baseline

T1: reading directly after application of tested materials

T2: reading after aging

The a* values of all groups decreased from T0 to T1, meaning a reduction in yellow or even a color shift towards green for the KI groups. This result became more accentuated in the SDF and GI groups but subsided in the CMP and KI groups with aging. However, KI resulted in the greenest values.

Immediately after the application of SDF, Group A showed a decrease in the b* value towards a less yellow color, and this value decreased even more from T1 to T2. In contrast, the b-values of KI and GI increased towards yellow from T0 to T1, and this yellow color subsided from T1 to T2. Regarding CMP, the b* value decreased directly after its application and then increased with aging to give a yellow color, which was less observable than the baseline reading.

## Discussion

The COVID-19 pandemic has drawn the attention of dental healthcare workers to the existing gap in the dental infection control standards implemented to prevent the transmission of airborne pathogens. Thus, SDF application is now recommended as one of the most important alternative nonaerosolizing management of dental caries [5, 6]. It supports an immediate and likely long-standing need to reduce aerosol-generating procedures in the dental therapy to minimize patient-to-patient transmission of SARS-CoV-2, to protect dental health care workers from harm, and to address in the long term a movement toward minimizing aerosol-generating procedures in dentistry, as required by Raskin et al. [5].

SDF has been extensively researched in children, and based on a recent systematic review by Jabin et al., the superiority of the effect of 38% SDF solution over 12% SDF solution and over placebos on primary dentition has been proven in vivo. Thus, SDF has been confirmed as one of the best treatment approaches in the control of dental caries in primary dentition [18]. However, at a 1-year follow-up, the survival rate of SDF treated teeth in patients aged 0 to 64 treated in community dental clinics was reported by Raskin et al. to have increased from 76 to 84% when covered by a restoration on the same day as SDF application [5]. This underlines the benefit that can be obtained by restoring teeth after SDF treatment and underscores the need for further research in this area.

Different restorative materials, such as self-cured GI (SCGI), resin-modified GI (RMGI) and CMP, have been used to restore cavitated teeth following SDF treatment (with or without KI). In their systematic review, Roberts et al. assessed the effectiveness and extent of staining reduction achieved when SDF was followed by KI application, showing that the application of KI after SDF might reduce staining [27]. Only one of the included studies, conducted by Nguyen et al., compared the impact of different white restorative materials on the lightness of tooth structures treated by SDF or SDF + KI [28]. However, the study was conducted on permanent teeth. Hence, its results cannot be generalized for primary teeth.

Color acceptance is a critical factor for the usage of any restorative or preventive material [21]. However, how to reduce the discoloration associated with the application of SDF in primary teeth remains unclear. Several studies have tried to measure the parental acceptance of SDF application in children [22]. One reported that only 53.3% of parents considered SDF treatment for their children acceptable due to discoloration problems [23]. Sabbagh et al. showed that parental acceptance of SDF treatment was significantly higher for posterior teeth than for anterior teeth [22]. Hence, this study aimed to quantify the color change produced by SDF in primary molars as well as the masking effect of the KI bleaching agent and CMP and GI restorations. Our study also followed up on these color changes after Suntest aging, which was shown to be a relevant method for testing discoloration [30].

This study is a laboratory trial, which limits the extrapolation of the results to the oral environment [27]. To prevent undesirable reactions between SDF and artificial saliva, as reported by Patel et al. [34], the specimens used in this study were stored in distilled water before the experiment, while in the experimental phase, dry specimens were used to show the absolute staining potential of SDF, meaning that the degree of staining in an oral environment may be slightly different from the results of our study due to the presence of saliva.

The randomization in our study was performed using the block technique, yet blinding was another limitation of our study [35]. Blinding was impossible since the color change caused by the SDF itself was evident. Moreover, the different compositions and colors of the added materials made them easily distinguishable from one another by the naked eye.

A spectrophotometer was used for the color measurement in this study, as it uses low light intensity to measure the full visible spectrum presented in the LAB system. This might have affected the reproducibility of the readings due to difficulties in positioning the sample exactly in the same place each time. A slight right or left shift of the sample can lead to changes in the lightness [36]. Therefore, 4 measurements were made for each reading, and their mean was used in the data analysis to minimize the percentage error.

This study was conducted on primary molars with carious lesions extending to the dentin of children aged 4–7 years of both sexes, suggesting that the results can be generalized to all dentinal lesions in primary molars.

The measured (ΔE) value did not provide enough information to interpret the color masking effect of the treatment groups due to a lack of expression of the direction of color change in the mathematical equation. However, the interpretation of the differences in the individual color parameters was more useful.

The color shift of SDF-treated dentin can be attributed to its chemical reaction, which suggests, as shown in Eq. (1), that SDF produces not only the free fluoride ions responsible for the remineralization of dentin and enamel but also a black silver precipitate causing discoloration [26].

1 $${\text{Ag}}({\text{NH3}}){\text{2F}}\left( {{\text{aq}}} \right) \to {\text{Ag}}\left( {\text{s}} \right) + {\text{2NH3}}\left( {\text{g}} \right) + {\text{F}} - \left( {{\text{aq}}} \right)$$

KI, as shown in Eq. (2), reverted the discoloration of SDF by reacting with the excess silver ions to produce silver iodide, which is yellowish in color and easily rinsed away with water [28].

2 $${\text{Ag}}({\text{NH3}}){\text{2F}}\left( {{\text{aq}}} \right) + {\text{KI}}\left( {{\text{aq}}} \right) \to {\text{AgI}}\left( {\text{s}} \right) + {\text{2NH3}}\left( {\text{g}} \right) + {\text{F}} - \left( {{\text{aq}}} \right)$$

Patel et al. reported no significant differences in gray values following SDF + KI application compared to baseline carious lesion values in primary molars [34]. In contrast, our results suggested a significant color shift of the KI group compared to the baseline color of the carious lesion, yet both studies found that KI can decrease the effect of staining caused by SDF. This difference in results may be attributed to either the differences in measuring techniques used, as Patel et al. used standardized time-lapse photography while we used spectrophotometry, or due to differences in the extent of caries and accordingly the baseline color of the carious lesions, since both studies used carious primary molars.

Changes in ΔL represented as the darkening of all treatment groups after aging could be explained by the photosensitivity of SDF. Zhao et al. reported that black metallic silver reproduction is accelerated by heat and light exposure, which was the case here after Suntest aging by a xenon lamp [37]. Lou et al. reported that silver iodide ions are photosensitive, which explains why the KI-treated group also showed darkening and discoloration with aging [38].

The L* value of CMP increased by aging in relation to the baseline reading; thus, CMP was the only material that maintained a color lighter than the tooth baseline color, which can be explained either by the increased color stability of CMP compared to that of conventional GI or by the more detrimental effect of SDF than of CMP on the L* scale of GI [39].

The difference in lightness (ΔL) between CMP and GI was not significant directly after their application, since both were the same shade (A1) and were subjected to light curing immediately after their application to polymerize either the CMP or the topcoat of GI. Hence, color changes due to light factors in the CMP and GI groups started directly after their application, while the other two groups exhibited such changes after aging. This finding was similar to the results in a systematic review by Roberts et al., which reported that restorative materials that require light curing, such as composites and resin-modified glass ionomers, show immediate grayish discoloration when applied after SDF. However, in contrast to our results, the color changes of those restorative materials were minimal over time, which can also be explained by the use of different aging methods independent of light sources [27].

A more in-depth analysis of ΔL revealed that GI produced a lighter tooth color directly after its application than did KI but resulted in a dramatic color change during aging that made GI less color stable than CMP and KI. This finding confirms the results of Hamama et al. [25], who stated that SDF can cause massive discoloration of GI restorations [25], with GI producing a darker color than both KI and CMP after aging.

Our results suggest that directly after their application, KI, CMP, and GI all had a satisfactory masking effect on the color change associated with the application of SDF, but the significant differences in the color shift after aging indicated that only one material was successful in masking the color change produced by SDF after aging: CMP.

According to our results, more studies should be conducted to test the ability of combinations of KI and GI/CMP to mask the color changes produced by SDF. Moreover, other GI alternatives, such as light-cured GI and reinforced GI, should also be tested.

## Conclusions

Within the limitations imposed by the experimental design used in this in vitro study, the following conclusions can be drawn:

1. Treatment with SDF caused obvious discoloration of carious dentin.
2. The factor “material” influenced the masking of the color change produced by the application of SDF on primary dentin.
3. Directly after SDF application, all materials (KI, CMP, and GI) could effectively mask the color change associated with the application of SDF.
4. CMP was the only material whose color masking effect was not completely reversed by aging.
5. Aging had a significantly different effect on the color shifts of all treatment materials and hence their masking effects, with the greatest effect being on GI and the least on CMP.
6. KI has good potential to mask the color changes associated with the application of SDF, but further studies are needed to test its masking effect in combination with CMP.

## Data Availability

The datasets used and/or analyzed during the current study are available from the corresponding author on reasonable request.

## Abbreviations

SDF: 38% Silver diamine fluoride solution
KI: Potassium iodide solution
CMP: Composite restoration
GI: Glass ionomer cement restoration
MANOVA: Multivariate analysis of variance
NRCT: Non-restorative cavity treatment
